# Perspektiven auf Schul- und Lebenswelten in Schüler/innen-Narrationen während der Corona-Krise im Frühjahr 2020

**DOI:** 10.1007/s35834-021-00299-2

**Published:** 2021-05-06

**Authors:** Wassilios Baros, Ulrike Greiner, Mishela Ivanova, Aida Delic

**Affiliations:** 1 FB Erziehungswissenschaft, Universität Salzburg, Erzabt-Klotz-Str. 1, 5020 Salzburg, Österreich; 2 School of Education, Universität Salzburg, Erzabt-Klotz-Straße 1, 5050 Salzburg, Österreich

**Keywords:** Schüler/innen-Sicht, Krisennarrationen, Fernunterricht, Krisenbewältigung von Schüler/innen, Schule in der Corona-Krise, Students’ perspectives, Crisis narratives, Home-schooling, Students’ processing the crisis, School in times of the Corona crisis

## Abstract

Ziel der internationalen Studie „*Futures Literacy* – Krisennarrationen von Kindern als Räume von Utopien der Solidarität“ ist es, über Krisennarrationen von Schüler/innen Einblicke in ihre Schulsituation und (Lebens‑)Welt in Zeiten der Corona-Krise zu gewinnen. Die Datenerhebung erfolgt auf der Basis eines im Rahmen von Schulaufgaben gegebenen Schreibauftrages an die Schüler/innen, bei dem diese darstellen sollten, wie sie in der fiktiven Zukunft (in 60 Jahren) in der Rolle der Großeltern ihren Enkelkindern von der Zeit der Corona-Krise erzählen. Angelehnt ist diese Vorgehensweise an das Konzept der Futures Literacy, welches Möglichkeiten eröffnet, mittels der Imagination von Zukunft Gegenwart besser zu verstehen und Handlungsoptionen zu entwickeln. Die besondere Schreibaufgabe soll helfen, herauszufinden, wie Schüler/innen aus verschiedenen europäischen Ländern mit der Corona-Krise umgehen, wie sie diese mit Blick auf Schule und häusliche Umwelt erleben und kognitiv und emotional verarbeiten und welche „Post-Corona“ Zukunftsperspektiven sie entwickeln. Die Auswertung erfolgt auf Basis einer Latent Class Analyse von 237 von den Schüler/innen produzierten Texte. Sie legt typische narrative und argumentative Textmuster offen, die zu bestimmten Narrationsstilen führen, aus denen sich die verschiedenen Perspektiven der Schüler/innen auf die Institution Schule, den Covid-19 bedingten Fernunterricht und den Lebensalltag rekonstruieren lassen, und die in spezifischen Zusammenhangskonstellationen mit ihrem Lebens- und Familienalltag sowie subjektiven Zukunftsbildern angesichts der Krise stehen.

## Abstract (en)

The aim of the international study “*Futures Literacy*—Children’s Crisis Narrations as Spaces of Utopias of Solidarity” is to gain insights into their (living) world and perspectives on home-schooling in times of the Corona Crisis by means of crisis narratives by schoolchildren. The data collection (*n* = 237) is based on a special writing assignment to the pupils, in which they are asked to describe how they tell their grandchildren about the time of the Corona crisis in the fictional future in the role of the grandparents—60 years later. The task requires an anticipatory effort from the present point of view, which stimulates them to reflect on their current situation from a certain “distance” (stimulating them to adopt a perspective and to construct a “vision”). The perspective of children is explicitly placed in the center of interest in order to draw conclusions about the issues that concern this age group in times of crisis. The aim is to find out how pupils from different European countries (Switzerland, Austria and Greece) deal with the Corona crisis and its consequences for their life-world, how they experience it and how they process it cognitively/emotionally. Theoretically, the study ties in with the concept of futures literacy. The texts produced by the students will be evaluated by means of a systematic combination of quantitative and qualitative content analysis (using Latent Class Analysis). Typical narrative and argumentative text patterns (narrative characteristics) will be identified.

Und dann durften wir nicht in die Schule gehen. Kannst du dir das vorstellen? (UP 1284, W)

## Problemstellung und Stand der Forschung

Aufgrund der Corona-Pandemie wurden im Frühjahr 2020 weltweit und auch in Europa Schulen für kurze oder längere Zeit geschlossen, wobei je nach Land oder Region hinsichtlich alternativer Formen der Schüler/innen-Betreuung, des Einsatzes von Lehr-Lern-Arrangements oder der Ferienregelungen sehr unterschiedlich vorgegangen wurde. In diesem Beitrag möchten wir Schüler/innen als Subjekte und Akteure von Schule ins Licht rücken, indem wir ihre Narrationen bezüglich der Corona-Zeit^1^ analysieren und ihre Perspektiven aufzeigen: Wie thematisieren Schüler/innen aus verschiedenen europäischen Ländern (Österreich, Deutschland, Schweiz, Griechenland) die Corona-Krise und die damit einhergehenden familiären, gesellschaftlichen und schulischen Veränderungen, wie erleben und bewerten sie diese und wie gehen sie mit der neuen Lebenssituation um?

Das Schulerleben sowie die subjektive Perspektive der Schüler/innen auf Schule, Bildung, Unterricht und gesellschaftliche Entwicklungen wird, anders als ihr objektivierbares Bildungsverhalten, selten in den Blick genommen. In den letzten Jahren ist der pädagogische Diskurs vielmehr durch die Betonung von leistungsbezogenen, fachlichen oder fachübergreifenden Ergebnissen oder gruppenspezifischen Vergleichen geprägt (vgl. z. B. Oberwimmer et al. 2019; Breit et al. 2019). Die Hervorhebung der lernenden Subjekte und ihr Blick auf die Institution Schule erfolgt nur selten (vgl. z. B. Eder 2007; Maschke und Stecher 2010; Bohnsack 2013) und meist verstärkt im Kontext qualitativer Studien (vgl. z. B. Hagedorn 2014).

Auch in Zusammenhang mit den durch die Corona-Pandemie und die damit einhergehenden Schulschließungen im Frühjahr 2020 veranlassten pädagogischen und erziehungswissenschaftlichen Studien, dominieren Erhebungen, welche die Schüler/innen meist als Betroffene und selten als Handelnde konzipieren. Ein großer Teil dieser Studien, viele in Form von Online-Befragungen, zielt darauf ab, die Situation im Frühjahr 2020 zu dokumentieren und die Rolle häuslicher und familiärer Merkmale für das Lernen von Schüler/innen zu beleuchten (Huber et al. 2020; Huber und Helm 2020).

So umfassend die Pandemie schulische Bildungsinstitutionen getroffen hat, so unterschiedlich sind die Konsequenzen auf den Lebens- und Schulalltag der betroffenen Schüler/innen (vgl. Hummrich 2020). Dementsprechend hat die Forschung begonnen, Aussagen zu differentiellen Effekten auf unterschiedliche Schüler/innengruppen zu generieren. Mittlerweile liegen Befragungen von Akteur/innen des Schulsystems, aber auch der Familienumwelten vor (etwa Huber et al. 2020; Vodafone Stiftung Deutschland 2020), welche die Bedingungen und Prozesse des ‚Distanzlernens‘ während der Corona-Krise bei unterschiedlichen Schüler/innengruppen in Abhängigkeit von ihren häuslichen Ressourcen und Selbstorganisationsfähigkeiten differenzieren. So kommen Huber und Helm (2020) auf der Grundlage der Daten des Schul-Barometers zu dem Ergebnis, dass Schüler/innengruppen aus sozioökonomisch schlechter gestellten bzw. benachteiligten Familien in Zeiten der Schulschließung insbesondere aufgrund fehlender Fähigkeiten zur Selbstorganisation, Tagesstrukturierung und zum selbstgesteuerten Lernen und weniger wegen fehlender technischer Ausstattung oder unzureichender elterlicher Unterstützung zurückbleiben.

In der Studie der Uni Wien „Lernen unter COVID-19-Bedingungen“ betonten die Schüler/innen ihre durch die Corona-Krise und den veränderten Unterricht gewonnene Einsicht in die Wichtigkeit der eigenen Lernorganisation. Zudem gaben sie an, dass sich ihr Wohlbefinden nach der Wiederöffnung erhöht hat, vor allem durch die wiedergewonnenen sozialen Kontakte (vgl. Schober et al. 2020).

Die bis jetzt abgeschlossenen Erhebungen bieten bereits erste Bestandsaufnahmen von Schüler/innen-Perspektiven auf die schulischen Auswirkungen der Krise^2^, die veränderte Schulorganisation und ihre Lernstrategien (vgl. auch Heller und Zügel 2020). Insbesondere bei den online stattgefundenen Befragungen wird allerdings ihre Stichprobenrepräsentativität kritisch diskutiert (vgl. Helm et al. 2020), da die technologisch nicht gut ausgestatteten Schüler/innengruppen möglicherweise systematisch nicht erreicht worden sind.

Aktuell liegen kaum Studien vor, die sich der Thematik mittels qualitativer oder Mixed-Methods-Ansätze nähern. Eine Ausnahme stellt die qualitative Befragung von Schüler/innen aus Baden-Württemberg dar (vgl. Wacker et al. 2020) oder die Studie SCHELLE, welche eine mehrperspektivische Befragung (Schüler-Eltern-Lehrerperspektive) zum sog. „Corona-Homeschooling“ im Mixed-Methods-Design vorgenommen hat (vgl. Letzel et al. 2020).

Während erste (vornehmlich quantitativ ausgerichtete) Studien zur Lernsituation von Schüler/innen im Kontext der Corona-Pandemie vorliegen, die auch die Dimension Wohlergehen einschließen, fehlen breitere erziehungswissenschaftliche Fragestellungen, die die Kognitionen, Emotionen und Bewertungen der Schüler/innen hinsichtlich der Krise und deren mögliche private und gesellschaftliche Auswirkungen in einen Zusammenhang mit dem Erleben und Beurteilen von Schule und Lernen in Krisenzeiten stellen.

## Das Forschungsprojekt „Futures Literacy – Krisennarrationen von Kindern als Räume von Utopien der Solidarität“

Im Rahmen der internationalen Studie „Krisennarrationen von Kindern als Räume von Utopien der Solidarität“ versuchen wir, über Krisennarrationen von Schüler/innen Einblicke in ihre subjektiven Betrachtungen der Corona-Krise zu gewinnen. Unser Ziel ist, herauszufinden, wie Schüler/innen aus verschiedenen europäischen Ländern mit der Corona-Krise umgehen, wie sie diese mit Blick auf die Institution Schule, die Gesellschaft und die häusliche Umwelt erleben und kognitiv sowie emotional verarbeiten und ob bzw. welche „Post Corona“ Zukunftsvorstellungen sie entwickeln.

**Abb. 1:**
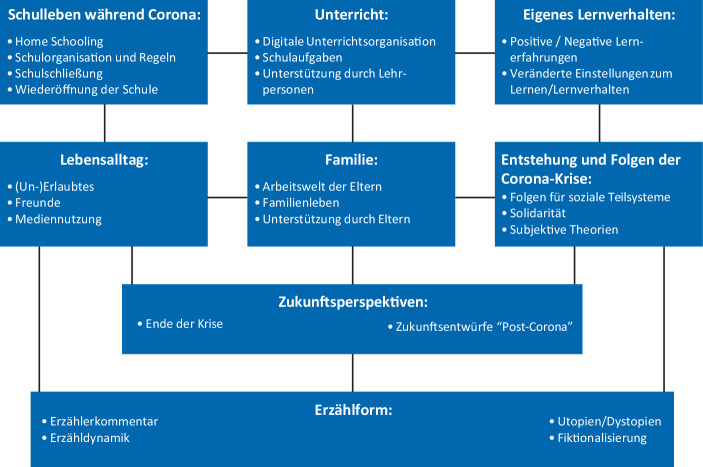
Heuristisches Rahmenmodell des Forschungsprojektes „Futures Literacy – Krisennarrationen von Kindern als Räume von Utopien der Solidarität“

Die oben angeführten Fragen stehen in der Tradition eines kontextualisierten, systemischen Ansatzes, bei dem Schule und Lernen (in der Corona-Zeit) im Kontext von schulüberschreitenden Konstellationen betrachtet werden. Die subjektive Erfahrung von Schule und Lernen wird mit Aspekten des Lebensalltags, der Familie und der Einschätzung der gesellschaftlichen Folgen der Krise und möglicher Zukunftsperspektiven zusammengesehen (vgl. Abb. 1).

Adressiert sind Jugendliche als Akteur/innen von Schule und zugleich als Subjekte gesellschaftlicher Krisenverarbeitung. Der Umgang mit Nicht-Wissen, zukunftsbezogener Ungewissheit und Ambiguität angesichts von Krisen – ebenso wie die Notwendigkeit, krisenhafte Ereignisse zu bewerten, Zukunftsszenarios zu imaginieren und zukunftsrelevante Entscheidungen zu treffen – wird als kognitive und psychosoziale Voraussetzung für „Futures Literacy“ (Miller 2007) genannt. Diese besondere Kompetenz basiert auf die Antizipation und Imagination von Zukunft sowie auf die Nutzung der Zukunftsvorstellungen. Unter der Annahme komplexer und interagierender Systeme geht es somit auch um das Verstehen und Lösen von Gegenwartsproblemen (Miller 2018).

## Präzisierung der Fragestellung

In der Studie wird angenommen, dass sich anhand der Rekonstruktion von kindlichen Narrationen jene mentalen Modelle der Krisenverarbeitung erfassen lassen, die die Situationsdefinitionen sowie Formen der Antizipation von Zukunft bei Schulkindern prägen. Somit stellt sich die Frage, welche mentalen Modelle in kindlichen Narrationen über die aktuelle Situation erkennbar sind, die auf unterschiedliche Formen der Wahrnehmung und kognitiven sowie emotionalen Verarbeitung der veränderten (schulischen) Realität schließen lassen?

Lassen sich in den Narrationen von Kindern typische Muster des Verstehens von Gegenwartsproblemen in ihrer Komplexität und der Antizipation von Zukunft identifizieren?

In welcher Weise prägen die gewählten Erzählformen die Aussagemöglichkeiten?

Wie wird die Erfahrung des Brüchigwerdens der Welt am Beispiel einer fundamentalen (gesellschaftlichen, ökonomischen) Krise mit einem Verständnis von intergenerationaler Solidarität verbunden?

Wie werden (Dis‑)Kontinuitäten in den kindlichen Lebenswelten durch das Spannungsverhältnis von digitalen und analogen Handlungskontexten beschrieben?

Dies sind Fragen, welchen wir im vorliegenden Forschungszusammenhang unserer Studie nachgehen möchten und welche sich auch in dem heuristischen Rahmenmodell widerspiegeln (vgl. Abb. 1), welches in der Konzeption der Gesamtstudie verankert ist und sich durch den Schreibauftrag in den Narrationen der Schüler/innen niederschlägt.

## Methodisches Vorgehen

### Untersuchungsverlauf und Datenmaterial

Um den Anspruch zu erfüllen, die Kognitionen, Emotionen und Bewertungen der Schüler/innen in einen Zusammenhang mit dem Erleben und Beurteilen von Schule und Alltag in Krisenzeiten zu stellen, greifen wir auf die Methode des „Youth Writing“ zurück. Schulische und außerschulische Initiativen zu Praktiken des Youth Writing dienen zuvörderst der Artikulation und Klärung eigener Einstellungen und Haltungen zu brennenden Themen des Lebensalltags, der Politik und Gesellschaft. Besonders im angelsächsischen Raum werden solche Praktiken als substantielle Gelegenheiten verstanden, Jugendlichen zuzutrauen, sich schreibend zu kontroversiellen Themen des gesellschaftlichen Alltags und der eigenen Identitätsentwicklung zu äußern (vgl. Hoechsmann und Lightman 2015). In unserer Studie kommt diese spezifische Schreibpraxis zur Anwendung, wie sie in der narratologischen Forschung oft mit Fokus auf Krisen und Krisenüberwindungen im Kontext der Antizipation möglicher Zukünfte eingesetzt wird (vgl. Balint und Wortmann 2020). Die Datenerhebung erfolgt auf der Basis eines besonderen Schreibauftrages an die Schüler/innen, bei dem diese darstellen sollen, wie sie in der fiktiven Zukunft (in 60 Jahren) in der Rolle der Großeltern ihren Enkelkindern von der Zeit der Corona-Krise erzählen:

Schreibaufgabe:

Es ist das Jahr 2080. Du bist über 70 Jahre alt und dein Enkel/deine Enkelin ist zu Besuch.

In den Medien wurde ausführlich über die Corona-Krise im Jahr 2020 berichtet. Dein Enkel/deine Enkelin ist sehr neugierig und möchte mehr wissen. Er/Sie fragt dich nach deinen Erinnerungen. Du erzählst ihm/ihr, wie alles begonnen hat, wie sich dein Alltag, dein Schulleben, dein Familienleben und der Kontakt zu deinen Freunden verändert hat.

Umfang 200–250 Wörter

Diesen Schreibauftrag erhielten die beteiligten Schüler/innen als unterrichtliche Schreibaufgabe von ihren Lehrpersonen, welche auch die Genehmigung der Eltern zur anonymisierten Verwendung der Texte für die Forschung einholten. Die angezielte Textsorte, eine schriftlich verfasste imaginierte Erinnerungsrede, thematisiert das Futur II als Schreibhaltung des „Es wird gewesen sein“. Die erzählte Welt ist dabei in der Gegenwart, die Erzählwelt in der Zukunft. Die Verschränkung von Gegenwart und Zukunft hat das Potenzial, ein utopisches und/oder dystopisches Denken und Schreiben zu stimulieren, je nachdem wie optimistisch oder pessimistisch man den Ausgang der Corona-Krise bewertet. Der indirekte Gegenwartsbezug gibt Auskunft über die subjektive Gegenwartseinschätzung. Das so entstehende Korpus von fiktiven Erzählungen einer „Großelterngeneration der Zukunft“ an ihre – noch ungeborenen – Enkel über das Leben in der Zeit der Corona-Krise dient der sozialwissenschaftlichen und erziehungswissenschaftlichen Forschung hier als Datenmaterial.

Im Rahmen der internationalen Studie „Krisennarrationen von Kindern als Räume von Utopien der Solidarität“ wurden zwei Erhebungswellen, während des 1. Lockdown (Frühjahr/Sommer 2020) und im Spätherbst/Winter 2020/21, geplant.

Die erste Erhebungswelle fand zwischen Mitte Mai und Anfang Juli 2020 statt – also in der Phase der allmählichen Wiederöffnung der Schulen. Dabei wurden 685 Schüler/innen im Alter zwischen 10 und 13 Jahren aus 16 Schulen in Österreich, Deutschland, Schweiz und Griechenland befragt. Die Wahl der Schulen und Regionen orientierte sich an bestehenden Kooperationen, die es ermöglicht haben, schnell zu agieren und zu Beginn der ersten Maßnahmen zur Eindämmung der Pandemie mit der Datenerhebung zu beginnen. Verschiedene Kontexte wurden aufgrund von unterschiedlichen Graden der Restriktion und Einhaltung von Quarantäne-Maßnahmen durch die Bevölkerung sowie im Fall Griechenland, aufgrund von kollektiven Erfahrungen mit Krisen^3^, als relevant erachtet.

Die Studie beschränkt sich auf die Gruppe der 10- bis 13-Jährigen, u. a., weil es in dieser Altersstufe möglich wird, Narrationen mit Perspektivenwechsel und kritisch-reflexiver Kommentierung zu entwickeln. Die gestellte Schreibaufgabe erfordert eine sich vom gegenwärtigen Standpunkt ergebende Antizipationsleistung, die dazu anregt, aus einer gewissen „Distanz“ heraus (Anregung zur Perspektivübernahme und zur Konstruktion eines „Weitblicks“) über die eigene aktuelle Situation zu reflektieren. Andererseits hat sich in dieser Altersgruppe noch kaum ein konventioneller Schreibstil im Sinne der Textsortenroutinen verfestigt.

Die Schüler/innen besuchten zum Zeitpunkt der Erhebung die vierte, fünfte oder sechste Schulstufe an einer Volksschule, Mittelschule oder an einem Gymnasium. Dieser Schultypen waren in Österreich als erste von den Schulschließungen ab März 2020 betroffen. Im Rahmen der ersten Erhebungswelle wurden Daten in den Bundesländern Salzburg, Tirol und Oberösterreich erhoben. Aus der Schweiz werden die Daten aus einer Oberschule aus St. Gallen berücksichtigt. Aus Deutschland nahmen eine Schule aus Berlin und eine aus Augsburg teil. Aus Griechenland fließen Daten aus einer Schule in Alexandroupolis und einer Schule in Volos ein.

Da keine ausgeglichene Variation zwischen den Regionen gewährleistet ist, kann eine regionale Repräsentativität der Stichprobe nicht beansprucht werden. Es ist allerdings davon auszugehen, dass im Rahmen der jeweiligen Schulen und Schultypen keine systematische Auslassung bestimmter Schüler/innen-Gruppen stattgefunden hat.

Zum Zeitpunkt der Erhebung hatten alle Schüler/innen die erste Phase des Lockdowns, der Schulschließungen, schon weitgehend hinter sich gebracht. Für die Erhebungen wurden ganze Klassen oder Teilgruppen einer Schulklasse hinzugezogen. In manchen Klassen oder Teilgruppen wurde der Schreibauftrag als Hausaufgabe erteilt, in anderen erfolgte die Textverfassung im Präsenzunterricht. Die klassenweise durchgeführte Erhebung hat Vor- und Nachteile. Einerseits konnten Stichprobenverzerrungen wie bei freiwilligen online-Befragungen vermieden werden, andererseits können Klassenzusammensetzungswirkungen und systematische Verzerrungen, verursacht durch die spezifischen Anleitungen der jeweiligen Lehrperson, nicht ausgeschlossen werden.

Neben Geschlecht, Alter, Schul- und Klassenzuordnung sowie Region (Stadt/Land) wurden keine weiteren soziodemographischen Variablen erhoben. Dadurch sollte vermieden werden, dass die Bearbeitung der Aufgabe kombiniert mit einem Fragenbogen den Charakter einer Befragung annähme, wodurch vermutlich die Kreativität der Schüler/innen bei der Bearbeitung des Aufsatzes eingeschränkt gewesen wäre.

Vor Beginn der systematischen Kodierung wurde überprüft, ob der für alle Schulen und Länder gleiche Schreibauftrag in unterschiedlichen kulturellen Kontexten und Sprachen (für die erste Teilstudie: Deutsch und Griechisch) ähnliche Dimensionen in den Texten auffindbar macht. Diesbezüglich kann man bestätigend von einem homogen funktionierenden Schreibimpuls ausgehen.

### Auswertungsmethode, Dimensionen und Variablen

Mit dem Ziel, Narrationsstile zu identifizieren, die Einblicke in die Schüler/innen-Perspektiven ermöglichen, wurden die von den Schüler/innen produzierten Texte mithilfe einer Latent Class Analyse (LCA) ausgewertet (vgl. Lazarsfeld 1950; Tarnai und Bos 1989; Baros und Kempf 2014). Es handelt sich dabei um ein Verfahren, das durch eine systematische Kombination von quantitativer und qualitativer Inhaltsanalyse die Identifikation typischer narrativer und argumentativer Textmuster erlaubt, welche die Rekonstruktion verschiedener, überindividuell geteilter Schüler/innen-Perspektiven ermöglichen. Die LCA erfüllt hierbei eine Brückenfunktion: Sie erlaubt, in großen Textkorpora enthaltene Klassen von Merkmalsmustern und zugleich typische Texte zu identifizieren, in welchen diese Merkmalsmuster in Reinform enthalten sind. Als typisch sind solche Texte zu betrachten, bei denen eine hohe Übereinstimmung zwischen den jeweiligen manifesten Textmerkmalen mit den latenten Merkmalen einer bestimmten Klasse besteht (membership probability, vgl. Kempf 2010). Diese können einer weiterführenden qualitativen Analyse unterzogen werden. Durch diese methodische Schrittfolge wird eine Validierung der Interpretation der quantitativen Ergebnisse (d. h. der latenten Klassen) ermöglicht und zugleich dem für quantitative Studien gängigen Anspruch auf Objektivierbarkeit, Repräsentativität und Generalisierbarkeit Genüge getan.

Das der folgenden Auswertung zugrundeliegende Datenmaterial setzt sich aus insgesamt 237 Texten zusammen, worunter ein Großteil aus Schulen in Österreich stammt (*n* = 183). Darüber hinaus sind Texte aus Griechenland (*n* = 37) und der Schweiz (*n* = 17) enthalten. Für diese Auswertung wurden Texte ausgewählt, deren Analyse einen Eindruck aus der ersten Phase der Corona-Krise gewährt. Texte aus Deutschland wurden dabei nicht berücksichtigt, da diese erst zu einem späteren Zeitpunkt eingegangen sind. Der Einbezug unterschiedlicher Kontexte beschränkt sich für diese explorative Studie daher auf die o. g. Auswahl.

Um in den Schüler/innen-Narrationen typische (latente) Muster zu identifizieren, wurden die Texte zuerst inhaltsanalytisch durch zwei dreiköpfige Teams untersucht. 54 dichotome Variablen wurden dabei induktiv aus den Aufsätzen abgeleitet und in fünf Dimensionen zusammengefasst (vgl. Tab. 1): I. Leitmotive und emotionale Verarbeitung (k = 14), II. Text-Erzählform (k = 8), III. Gegenwarts- und lebensweltüberschreitendes Denken (k = 10), IV. Schul- und Unterrichtsorganisation (k = 15) und V. Erleben von Schule und Lernverhalten (k = 7). Im Anschluss an die induktive Kategorienbildung wurden alle Texte entlang der 54 Variablen kodiert. Die Operationalisierung durch binäre Variablen (0 = Merkmal kommt nicht vor; 1 = Merkmal kommt vor) erweist sich insofern als besonders günstig, als sich die an den Text gerichteten Fragen vergleichsweise einfach und intersubjektiv nachvollziehbar beantworten lassen. In Tab. 1 sind alle 54 Variablen dargestellt und erläutert.

**Tab. 1:** Definition der inhaltsanalytischen Variablen (Dimensionen I–V)

Variable	Definition
**Dimension I:** Leitmotive und emotionale Verarbeitung	
1	*Schulleben während „Corona“:* Die Auswirkungen der Corona-Krise auf das Schulleben werden thematisiert
2	*Veränderungen im Alltag:* Veränderungen, die den Alltag der Schüler/innen oder der Bevölkerung betreffen, werden thematisiert
3	*Familiäre Situation und Veränderungen in der Arbeitswelt der Eltern:* Die Auswirkungen einer Veränderung in der Arbeitswelt der Eltern während der Corona-Krise auf das Familienleben werden beschrieben
4	*Freundschaften/Peers:* Der Kontakt der Schüler/innen zu Freunden und sozialen Netzwerken wird im Zusammenhang mit der Corona-Krise thematisiert
5	*Medien und Digitalisierung:* Die Verwendung/Nutzung von (sozialen, digitalen) Medien wird im Zusammenhang mit der Corona-Krise genannt oder beschrieben
6^a^	*Subjektive Theorien:* Es werden Erklärungen bezüglich der Entstehung/Verbreitung/Auswirkung des Corona-Virus formuliert
7	*Wissenschaftliche/medizinische Informationen:* Der Text enthält wissenschaftliche und medizinische Informationen und/oder Darstellungen des Corona-Virus, die die Entstehung und die Auswirkungen des Virus näher erläutern
8^a^	*Covid-Akut:* Über Corona-Fälle in der Familie, im Verwandtschaftskreis oder in der Nachbarschaft wird berichtet
9^a^	*Körperlichkeit:* Äußerungen im Zusammenhang mit Bewegung und Körper werden gemacht
10^a^	*Unerlaubtes:* Es wird auf nicht gestattete Aktivitäten eingegangen
11^a^	*Erlaubtes:* Es wird auf gestattete Aktivitäten eingegangen
12^a^	*Krisenfolgen positiv beurteilt:* Die Corona-Krise, die Krisenfolgen und der Prozess der Krisenbewältigung werden von der Erzählung als überwiegend utopisch dargestellt
13^a^	*Krisenfolgen negativ beurteilt:* Die Corona-Krise, die Krisenfolgen und der Prozess der Krisenbewältigung werden von der Erzählung als überwiegend dystopisch dargestellt
14^a^	*Raumeinschränkung/räumliche Isolierung wird explizit thematisiert:* Die sozialgeographische Beschränkung der eigenen Lebenswelt und/oder der Lebenswelten der Menschen wird thematisiert
**Dimension II: **Text-Erzählform	
15	*Formale Fiktionalisierung – Ansprache:* Die Erzählung ist als Erzähl-Szene zwischen Personen im Sinn des Schreibauftrags gestaltet (wobei charakteristisch „mündliches“ Erzählen überwiegt, die Zuhörer/innen werden adressiert, z. B. das Enkelkind wird in den Schilderungen einleitend konkret angesprochen)
16	*Formale Fiktionalisierung – Kontext:* Der Text beinhaltet eine räumliche Kontextualisierung der Erzählsituation
17	*Bewertungen der Corona-Situation/Erzählreflexion:* Die narrativ dargestellten Ereignisse werden durch explizite Erzählerkommentare perspektiviert bzw. evaluiert
18	*Erzähldynamik dynamisch:* Die mit Coronaausbruch und Lockdown verbundenen Ereignisse werden mit Orientierung auf Abfolge von Ereignissen (z. B. durch Verwendung von zeitlichen Wörtern, die einen Prozess nachzeichnen) erzählt
19	*Erzähldynamik statisch:* Die mit Coronaausbruch und Lockdown verbundenen Ereignisse werden mit Orientierung auf Gleichzeitigkeit von Zuständen erzählt, wobei eine zeitliche Abfolge in der Erzählung nicht erkennbar ist
20	*„Vor-Corona“:* Die jeweiligen privaten und/oder gesellschaftlichen Zustände vor der Corona-Krise sind im Text realisiert
21	*„Post-Corona“/Zukunftsentwurf:* Die Zeit nach dem Schreibzeitpunkt (also die Zeit zwischen 2020 und „2080“) wird vom Text als Periode mit besonderen Charakteristika realisiert
22	*Verallgemeinerung:* Zusätzlich zur Ich‑/Wir-Perspektive wird auch ein Standpunkt mit Schilderung des allgemeinen Geschehens (z. B. „die Menschen“, „Die Schulen“) eingenommen
**Dimension III:** Gegenwarts- und lebensweltüberschreitendes Denken	
23	*Corona als globale räumliche Dimension:* Über die eigene Lebenswelt hinaus werden größere sozialgeographische Dimensionen genannt: Nachbarländer, weltweite Perspektive, territoriale Ausdehnung des Virus etc
24	*Epistemologische Überschreitung, Fiktionalisierung:* Es wird eine alternative (fiktionale) mögliche Welt entworfen, nicht nur im Sinne einer formalen Perspektive, sondern tatsächlich im Sinne einer fiktiven Welt
25	*„Wir und Andere“:* Bei der Thematisierung des Betroffen-Seins durch den Virus werden Grenzziehungen zwischen „Wir“ und „Anderen“ vorgenommen
26	*Thematisierungen weiterer sozialer Probleme:* Es werden weitere soziale Probleme, die im Kontext von Corona stehen können, aber nicht müssen, thematisiert
27	*Wirtschaftskrise:* In den Texten wird Wirtschaftskrise als Folge der Corona-Krise explizit benannt
28	*Bleibende Veränderungen seit „Corona“:* Nach Corona ist die Welt eine andere, entweder weil der Corona-Zustand immer noch andauert und/oder nicht mehr vergehen wird
29	*Ende der Krise:* In den Beschreibungen, die sich auf die Zeit nach Corona beziehen, wird von einem Ende der Krise ausgegangen. Die Corona-Krise erscheint als ein Zustand, der als abgeschlossen gilt
30	*Krise als Dauerzustand:* Covid-Zustand immer noch präsent. Dauerkrisen seit dieser Zeit; Krise wird entweder als Bedrohungsszenario oder als Herausforderung thematisch
31	*Folgen von Corona für andere Teilsystemen der Gesellschaft:* „Corona“ ist kein isoliertes (medizinisches, hygienisches etc.) Problem; es tangiert andere gesellschaftliche Bereiche, die entweder im Kontext Corona entstanden sind oder extra bestehen, und jetzt damit zusammengesehen und zusammengedacht werden
32	*Solidarität als Krisenbewältigung:* Äußerung des Wunsches nach mehr Solidaritätsbewusstsein für die zukünftigen Generationen
**Dimension IV:** Schul- und Unterrichtsorganisation	
33	*Digitale Schulorganisation:* Teilnehmer/in äußert, dass die Corona-Krise die Art und Weise der Hausaufgaben verändert hat
34	*Schulschließung:* Der Text enthält subjektive Bewertungen der Schulschließung
35	*Pro Schulschließung:* Positive Emotionswörter in Zusammenhang mit der Schulschließung werden geäußert
36	*Contra Schulschließung:* Negative Emotionswörter in Zusammenhang mit der Schulschließung werden geäußert
37	*Art der Aufgaben:* Die Umstellung auf neue Arten von Schulaufgaben wird thematisiert
38	*Digitaler Unterricht:* Die veränderte (digitale) Form des Unterrichts wird erwähnt
39	*Wiedereröffnung:* Die Situation nach der Wiedereröffnung der Schule wird geschildert
40	*Schulregeln:* Die Arbeitsorganisation in der Schule während des digitalen Fernunterrichts wird beschrieben
41	*Verwunderung:* Die Tatsache, dass die Schulen schließen mussten, wird als etwas merkwürdiges dargestellt; Verwunderung wird geäußert
42	*Unterstützung/Lehrpersonen:* Die unterstützende Rolle von Lehrpersonen während dieser Zeit wird hervorgehoben
43	*Fehlende/Unzureichende Unterstützung von Lehrpersonen:* Die fehlende bzw. unzureichende Unterstützung von Lehrpersonen während dieser Zeit wird moniert
44	*Pro-Wiedereröffnung:* Positive Emotionen in Zusammenhang mit der Wiedereröffnung der Schulen werden artikuliert
45	*Kontra-Wiedereröffnung:* Negative Emotionen in Zusammenhang mit der Wiedereröffnung der Schulen werden artikuliert
46	*Unterstützung/Eltern:* Die unterstützende Rolle der Eltern während dieser Zeit wird hervorgehoben
47	*Nicht-Unterstützung/Eltern:* Die fehlende oder unzureichende Unterstützung der Eltern während dieser Zeit wird moniert
**Dimension V: **Erleben von Schule und Lernverhalten	
48	*Schulaufgaben:* Schulaufgaben werden als belastend bezeichnet
49	*Arbeitsüberladung:* Es wird die große Menge der Schulaufgaben kritisiert
50	*Rückblick auf Schule:* Schule wird in den Schilderungen der Kinder explizit aus einer Zukunftsperspektive kommentiert
51	*Lernverhalten (neutral):* Auf das eigene Lernverhalten während der Corona-Zeit wird eingegangen
52	*Lernverhalten (positiv):* Das eigene Lernverhalten während der Corona-Zeit wird positiv bewertet
53	*Lernverhalten (negativ):* Das eigene Lernverhalten während der Corona-Zeit wird negativ bewertet
54	*Veränderte Einstellungen über Schule und/oder Lernen:* Verweise auf veränderte Einstellungen über Schule und/oder Lernen oder es wird über verändertes Lernverhalten berichtet

^a^Variablen, die der Teildimension „emotionale Verarbeitung der Krise“ angehören

**Abb. 2:**
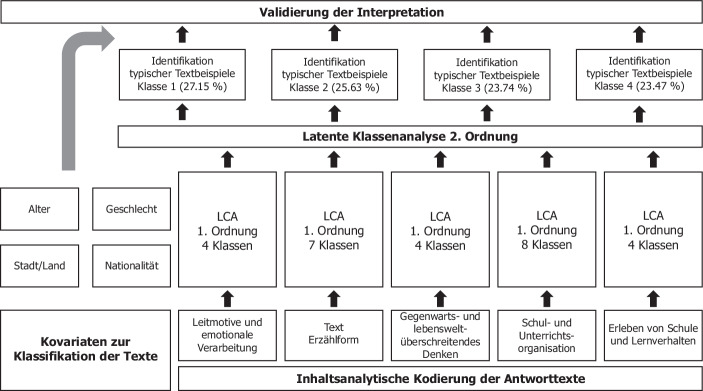
Design der Datenauswertung

Zu jeder der fünf Dimensionen wurde eine Latente Klassenanalyse (LCA) (Tarnai und Bos 1989; Baros und Kempf 2014) durchgeführt^4^^,^^5^. Auf die Darstellung der einzelnen gemäß AIC identifizierten Klassen der LCA 1. Ordnung wird an dieser Stelle aus Platzgründen verzichtet, zumal die einzelnen Klassen als Kovariaten im Rahmen der LCA 2. Ordnung berücksichtigt werden (vgl. Abb. 2). In der LCA 2. Ordnung wird untersucht, wie die fünf Dimensionen (A. Leitmotive und emotionale Verarbeitung, B. Text-Erzählform, C. Gegenwarts- und lebensweltüberschreitendes Denken, D. Schul- und Unterrichtsorganisation und E. Erleben von Schule und Lernverhalten), miteinander interagieren. Durch Kontingenzanalysen wurden anschließend mögliche Effekte der Kovariaten Alter und Geschlecht der Schüler/innen, sowie Region (Stadt/Land) und Nationalität überprüft. Zur Validierung der Interpretationen werden exemplarisch typische Passagen aus den Texten vorgestellt.

## Latent Class Analyse 2. Ordnung – Ergebnisse und Interpretationen

Im Folgenden werden als Ergebnis der LCA 2. Ordnung latente Narrationsstile von Schüler/innen skizziert. Es wird davon ausgegangen, dass sich in Textnarrationen mentale Repräsentationen manifestieren, deren Rekonstruktion die Formulierung erster Hypothesen bezüglich der Wahrnehmung von und des Umgangs mit Umwälzungen in der eigenen Lebens- und Schulwelt zulässt. Die Variablen dieser LCA 2. Ordnung sind in Tab. 2 dargestellt.

**Tab. 2:** Variablen der Latent Class Analyse zweiter Ordnung

Item	Kodierung
*A* Leitmotive und emotionale Verarbeitung	0 = Kl. 1 (56,36 %): Starke Wahrnehmung von Umwälzungen
	1 = Kl. 2 (21,15 %): Veränderungen im Alltag, medizinische Informationen, subjektive Theorien
	2 = Kl. 3 (11,71 %): Deskriptive Schilderungen von Veränderungen
	3 = Kl. 4 (10,77 %): Kaum Einschränkungen in der eigenen Lebenswelt
*B* Text-Erzählform	0 = Kl. 1 (42,71 %): Fokus auf gegenwärtige Situation, dynamische Erzählweise, keine echte Perspektivübernahme
	1 = Kl. 2 (13,73 %): Fokus auf gegenwärtige Situation, statische Erzählweise, keine Perspektivübernahme
	2 = Kl. 3 (11,87 %): Deutliche Gegenwartsüberschreitung, dynamische Erzählweise, Perspektivübernahme
	3 = Kl. 4 (11,08 %): Häufig Gegenwartsüberschreitung, dynamische Erzählweise, nicht selten Perspektivübernahme
	4 = Kl. 5 (9,91 %): Nicht selten Gegenwartsüberschreitung, ausgeprägte Fähigkeit zur Perspektivübernahme
	5 = Kl. 6 (5,93 %): Fokus auf gegenwärtige Situation, statische Erzählweise, häufig echte Perspektivübernahme
	6 = Kl. 7 (5,77 %): Kaum Gegenwartsüberschreitung, statische Erzählweise, keine echte Perspektivübernahme
*C* Gegenwarts- und lebensweltüberschreitendes Denken	0 = Kl. 1 (43,49 %): Hoffnung auf ein Ende der globalen Corona-Krise
	1 = Kl. 2 (34,09 %): Ausblendung der globalen Dimension und der gesellschaftlichen Folgen der Krise, Hoffnung auf ein Ende der Krise
	2 = Kl. 3 (12,88 %): Reflexion politischer und sozialer Folgen, Glauben an eine solidarische Überwindung der Corona-Krise
	3 = Kl. 4 (9,53 %): Krise als Dauerzustand mit bleibenden Veränderungen, Tendenz zur Fiktionalisierung
*D* Schul- und Unterrichtsorganisation	0 = Kl. 1 (20,2 %): Pro u. Contra Schulschließung
	1 = Kl. 2 (14,8 %): Gelegentliche Nennung der Lernumwelt, keine Positionierungen
	2 = Kl. 3 (13,79 %): Detaillierte Schilderung der neuen Schul‑/Unterrichtsorganisation, kaum Positionierungen
	3 = Kl. 4 (11,98 %): Detaillierte Schilderung der neuen Schul‑/Unterrichtsorganisation, starke Positionierungen: Pro u. Contra Schulschließung, Pro Wiedereröffnung
	4 = Kl. 5 (11,36 %): Detaillierte Schilderung der neuen Schul‑/Unterrichtsorganisation, Pro Wiedereröffnung, Pro u. Contra Schulschließung
	5 = Kl. 6 (11,1 %): Digitale Schulorganisation, digitaler Unterricht, Pro u. Contra Schulschließung
	6 = Kl. 7 (8,86 %): Detaillierte Schilderung der neuen Schul‑/Unterrichtsorganisation (insb. digitale Schule u. Schulschließung), Contra Schulschließung, Pro u. Contra Wiedereröffnung, (fehlende) Unterstützung durch Lehrer/innen u. Eltern
	7 = Kl. 8 (7,92 %): Schulschließung, Wiedereröffnung, Schulregeln, Pro u. Contra Schulschließung, Pro Wiedereröffnung
*E* Erleben von Schule und Lernverhalten	0 = Kl. 1 (55,84 %): Ausgeglichene Arbeitsbelastung, kaum Thematisierung von Lernverhalten
	1 = Kl. 2 (25,73 %): Generell intensives Empfinden von Arbeitsbelastung, neutrale Nennung des eigenen Lernverhaltens
	2 = Kl. 3 (11,0 %): Situationsbedingte hohe Arbeitsbelastung, Bewertung des eigenen Lernverhaltens
	3 = Kl. 4 (7,42 %): Positives Erleben der schulischen Situation, relativ häufige Thematisierung von Arbeitsbelastung

Die Analyse ergab laut AIC (Akaike 1987) eine 4‑Klassen-Lösung (vgl. Tab. 3), welche mit einem Proportional Reduction of Error von PRE = 53,95 % und einer mittleren Zuordnungswahrscheinlichkeit von MEM = 90,19 % eine zufriedenstellende Modellanpassung aufweist.

**Tab. 3:** Goodness-of-Fit-Statistiken der LCA 2. Ordnung (*n* = 237; m = 4; k = variabel)

Modell	ln(L)	*n*(P)	df	L‑Ratio	*p*	AIC	BIC
**LC1**	−1679	22	3561	916,7	n. b.	3402	3478
**LC2**	−1614	45	3538	787,92	n. b.	3319	*3475*
**LC3**	−1581	68	3515	720,8	n. b.	3298	3533,7
***LC4***	*−1552*	*91*	*3492*	*662,28*	*n.* *b.*	*3285*	3600,9
**LC5**	−1533	114	3469	624,68	n. b.	3294	3689,1
**LC6**	−1516	137	3446	591,14	n. b.	3306	3781,3
**LC7**	−1505	160	3423	568,06	n. b.	3329	3884
**Saturiert**	−1221	3583	–	–	–	9607	22033

**Abb. 3:**
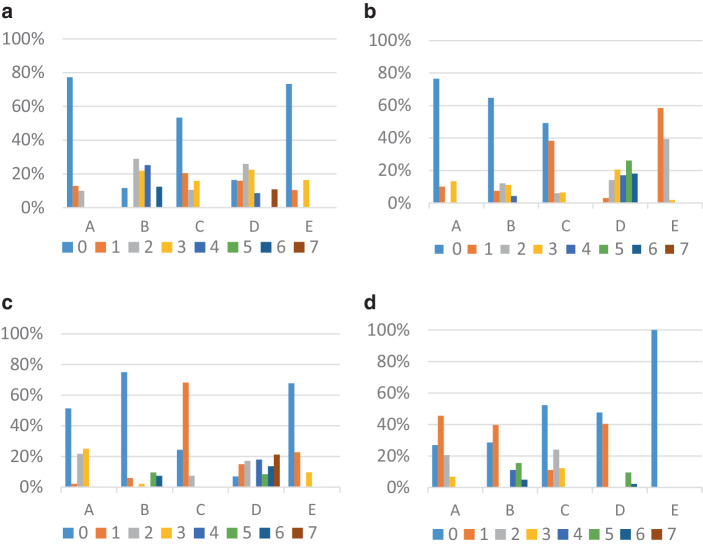
Latent Class Analyse zweiter Ordnung: Latente Narrationsstile. Zur Definition der Variablen vgl. Tab. 2. **a** Klasse 1: 27,15 %. Latenter Narrationsstil 1: Überschreitendes Denken und Tendenz zur Fiktionalisierung. **b** Klasse 2: 25,63 %. Latenter Narrationsstil 2: Starke Wahrnehmung der Diskrepanz zwischen analoger und digitaler Welt. **c** Klasse 3: 23,74 %. Latenter Narrationsstil 3: Distanziertes Wahrnehmen von Krise und Veränderungen im Schulalltag. **d** Klasse 4: 23,47 %. Latenter Narrationsstil 4: Sensibilität gegenüber sozialen Problemen und wenig selbstreflexiv in Bezug auf Schule

**Abb. 4:**
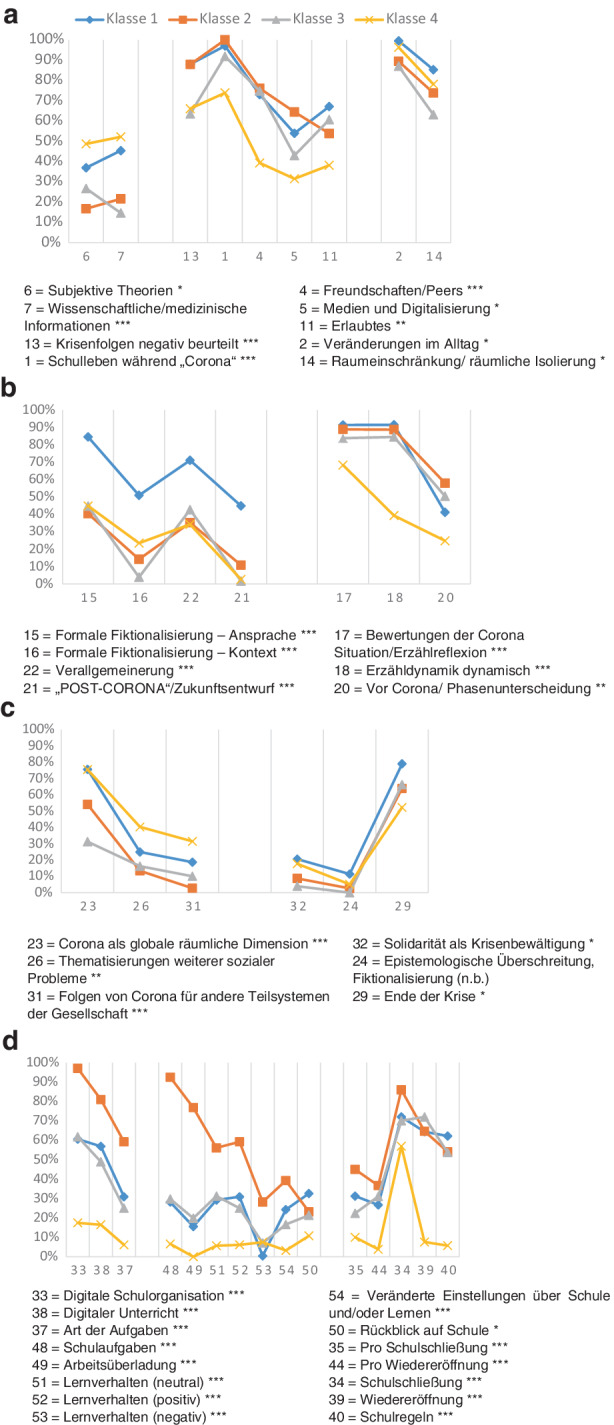
Latent Class Analyse zweiter Ordnung: Häufigkeiten signifikanter Textmerkmale Dimension I bis V (Chi-Quadrat-Test; *p < 0,01, **p < 0,005, ***p < 0,001). **a** Leitmotive und emotionale Verarbeitung. **b** Text- und Erzählform. **c** Gegenwarts- und lebensweltüberschreitendes Denken. **d** Schul- und Unterrichtsorganisation – Erleben von Schule und Lernverhalten

Die Charakteristika der identifizierten Klassen sind in Abb. 3 dargestellt, und die Abb. 4a–d zeigen die Häufigkeiten der zur Interpretation der Klassen herangezogenen Ausgangsvariablen.

**Abb. 5:**
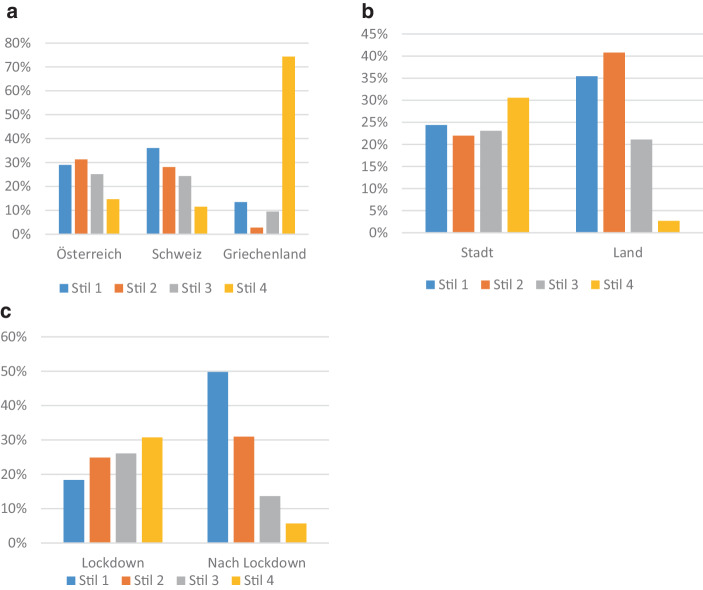
Relative Häufigkeiten der Stile in Abhängigkeit von **a** den Ländern (Chi-Quadrat = 63,41, *p* < 0,001, df = 6), **b** Stadt/Land (Chi-Quadrat = 22,23, *p* < 0,001, df = 3) und **c** Zeitpunkt der Erhebung (Chi-Quadrat = 33,81, *p* < 0,001, df = 3)

Texte, die noch während des ersten Lockdowns verfasst wurden, sind tendenziell eher in Stil 4, während später verfasste Texte eher in den anderen Stilen, insbesondere im latenten Stil 1 vorzufinden sind. Die Nationalität erweist sich als signifikant für die Zuordnung der Texte zu den Klassen. Dabei ist z. B. der Narrationsstil 4 für Texte aus Griechenland, der Narrationsstil 1 eher für Texte aus der Schweiz charakteristisch. Texte aus ländlichen Regionen sind tendenziell eher in den Stilen 1 und 2 zu finden, wobei Texte aus städtischen Region eher in Stil 4 wiederzufinden sind (vgl. Abb. 5). Die Variablen Alter (Chi-Quadrat = 1,21, df = 3, n. s.) und Geschlecht (Chi-Quadrat = 5,15, df = 3, n. s.) sind nicht signifikant.

Im Folgenden werden die Ergebnisse der Latenten Klassenanalyse (LCA) nach Klassen dargestellt. Die Darstellung der Veranschaulichungsbeispiele erfolgt unter Angabe der s. g. *Membership Probabilities*, die für jeden Text und jede Klasse die Wahrscheinlichkeit angeben, mit welcher die manifesten Merkmale des jeweiligen Textes mit dem latenten Merkmalsmuster der Klasse übereinstimmen.

### Klasse 1 (27,15 %): Überschreitendes Denken und Tendenz zur Fiktionalisierung

Charakteristisch für diesen latenten Narrationsstil sind Fiktionalisierungen, sowohl formal und gegenwartsüberschreitend (Post-Corona/Zukunftsentwurf 44,8 %) als auch im Sinne des Entwurfs alternativer (fiktionaler) Welten (11,5 %). Häufig und deutlich stärker im Vergleich zu den anderen Narrationsstilen werden hier Erzählkontexte entworfen, die auf eine Fähigkeit zur Antizipation und Perspektivübernahme hindeuten. Diese Klasse ist auch diejenige, die am häufigsten bleibende Veränderungen seit Corona (7,8 %) und die Krise als Dauerzustand (17,3 %) thematisiert und gleichzeitig an das Solidaritätsbewusstsein für die zukünftigen Generationen als Krisenlösungsstrategie (20,6 %) appelliert. Im Vergleich zu den anderen Klassen wird hier Schule am häufigsten explizit aus einer Zukunftsperspektive kommentiert (32,6 %). Dabei werden die mit der digitalen Dimension von Lebens- und Lern(um)welten einhergehenden Veränderungen mit häufigem Verweis auf Regeln der Arbeitsorganisation in der Schule während des digitalen Fernunterrichts (62,2 %) und mit positiver Selbsteinschätzung hinsichtlich des eigenen Lernverhaltens (30,9 %) geschildert. Es fällt auch auf, dass sich in dieser Klasse so gut wie keine negativen Thematisierungen des eigenen Lernverhaltens finden.

An einem verregneten Sonntagnachmittag kam meine Enkeltochter Sarah zu Besuch. Wir spielten schon die dritte Runde Mensch Ärgere dich nicht als sie plötzlich Hunger bekam. Also ging ich in die Küche, um ein paar Wurstbrote zu machen. Die Kleine durfte inzwischen fernsehen. Als ich wieder kam war Sarah ganz aufgeregt, sie rief mir schon von weitem entgegen: „Oma, Oma was ist Corona?“ Ich begriff sofort, es muss ein Bericht darüber gewesen sein. Natürlich erinnerte ich mich gleich an diese ungewöhnliche Zeit und begann zu erzählen. […] „Aber was war mit der Schule, Oma?“ Die Schulen hatten auch alle geschlossen. Wir hatten Homeschooling (UP 1006, MP 99,7 %, W)^6^.

### Klasse 2 (25,63 %): Starke Wahrnehmung der Diskrepanz zwischen analoger und digitaler Welt

Bei diesem Narrationsstil werden die Krisenfolgen häufiger als bei den anderen Klassen negativ beurteilt (87,8 %), wobei fast immer auch die direkten Veränderungen des Schullebens (99,9 %) thematisiert werden. Kennzeichnend für diesen Narrationsstil ist zudem der Fokus auf Veränderungen im Vergleich zu der Zeit vor der Krise: Die Verwendung/Nutzung von (sozialen, digitalen) Medien wird im Zusammenhang mit der Corona-Krise genannt (64,5 %) und die Konsequenzen der Krise auf die sozialen Beziehungen und Kontakte im Alltag (Freundschaften/Peers) (76,0 %) beschrieben. Dieser Narrationsstil zeichnet sich darüber hinaus aus durch eine überproportionale Thematisierung der Institution Schule (z. B. die Veränderung der Hausaufgaben 97,1 %, subjektive Bewertungen der Schulschließung 85,9 %, Artikulation von positiven Emotionswörtern im Zusammenhang mit der Schulschließung 45 %), der Unterrichtskultur (digitale Form des Unterrichts 81 %, große Menge der Schulaufgaben 76,8 %, Umstellung auf neue Art von Schulaufgaben 92,5 %) und des eigenen Lernverhaltens während der Corona-Krise – entweder positiv (59,3 %) oder auch negativ (28,2 %) bewertet. Insgesamt verweist dieser Narrationsstil auf veränderte subjektive Einstellungen zu Schule und/oder Lernen bzw. Lernverhalten (39,23 %). Themen wie soziale Probleme (16,3 %) oder Folgen für weitere Teilsysteme der Gesellschaft (2,8 %) sind in diesem Narrationsstil dagegen kaum vertreten. Als lebensweltüberschreitendes Thema ist lediglich Corona in seiner globalen räumlichen Dimension (54,1 %) realisiert. Die Texte können zu 37,5 % der ländlichen Region zugeordnet werden.

Kurze Zeit später wurden die Schulen geschlossen und wir bekamen über Portal Tirol die ganzen Hausübungen auf. Über zwei Monate mussten wir mit dem Computer arbeiten, das oft sehr anstrengend und ungewohnt war. Ungefähr zehnmal hatte unsere Klasse mit unseren Klassenvorständen Videokonferenz. […] Leider durfte man sich in dieser Zeit nicht mit Freunden treffen. Daher telefonierte ich jeden Tag mit ihnen oder machte Videochat (UP 1010, MP 100 %, W).

### Klasse 3 (23,74 %): Distanziertes Wahrnehmen von Krise und Veränderungen im Schulalltag

Dieser latente Narrationsstil enthält im Vergleich zu den anderen Klassen weniger häufig negative Beurteilungen der Corona-Krise, der Krisenfolgen und der Krisenbewältigung (66,1 %). Die Thematisierung eines veränderten Alltags (87,0 %) und der sozialgeographischen Beschränkung der eigenen Lebenswelt (63,1 %) fällt ebenfalls geringer aus. Im Mittelpunkt steht vielmehr die Thematisierung der Situation nach der Wiedereröffnung der Schule (72,0 %). Andere Veränderungen betreffend den Schulalltag (z. B. veränderte Einstellung über Schule oder Lernen 16,6 %) oder die Schulorganisation während der Krise (Art der Aufgaben 25 %, Schulregeln 53,7 %) geraten in den Hintergrund. Über die eigene Lebenswelt hinaus werden größere sozialgeographische Dimensionen (31,3 %) relativ deutlich und weitere soziale Probleme (16,3 %) sowie die Folgen für andere Teilsysteme der Gesellschaft (10,1 %) nur gelegentlich thematisiert. Die Schilderungen enthalten so gut wie nie Entwürfe alternativer (fiktionaler) Welten, räumliche Kontextualisierungen der Erzählsituation (0,3 %) oder Zukunftsentwürfe (2,6 %). Diese Texte sind an der Wiederkehr „normaler“ Verhältnisse orientiert, indem v. a. das Ende der Krise (66,3 %) und die Wiedereröffnung (72,0 %) fokussiert werden. Die Corona-Krise erscheint in diesem latenten Narrationsstil lediglich als „Zwischenfall“ ohne langfristige Folgen für die eigene Lebenswelt oder die Gesellschaft.

Als die Zeit gekommen war, in der wir mit den Masken einkaufen gehen mussten, bin ich gleich mal zu einem Geschäft gefahren. Ich habe mich nämlich schon vorbereitet wieder in die Schule zu gehen, aber es hat ja noch eine Weile gedauert, bis das wir wieder in die Schule konnten. Als wir wieder in die Schule kommen durften, mussten wir Masken tragen und auch die Hände immer sauber halten. Wenn man krank war, musste man zuhause bleiben. Am Platz konnte man die Masken abnehmen, wenn, man aufstand musste man sie wieder aufsetzen. Als uns mitgeteilt wurde, dass wir ohne Maske wieder in die Schule durften, war ich sehr froh (UP 1345, MP 99,9 %, M).

### Klasse 4 (23,47 %): Sensibilität gegenüber sozialen Problemen und wenig selbstreflexiv in Bezug auf Schule

Dieser latente Narrationsstil zeichnet sich dadurch aus, dass häufig Erklärungen bezüglich der Entstehung, Verbreitung und Auswirkung des Corona-Virus (48,7 %) formuliert werden und Faktenwissen (52,2 %) angeführt wird. Die Krisenfolgen werden vergleichsweise in geringerem Ausmaß negativ beurteilt (66,1 %) und Ausführungen über die Änderungen in der eigenen Lebenswelt (Schulleben, Freundschaften/Peers, Medien und Digitalisierung, Erlaubtes) fallen schwächer als bei den anderen Klassen aus. Berichte über Veränderungen im Alltag und die räumliche Isolierung sind fast immer angeführt. Texte dieser Klasse weisen am wenigsten Phasenunterscheidungen auf: Die jeweiligen privaten und/oder gesellschaftlichen Zustände vor (24,8 %) und nach (2,6 %) der Corona-Krise sind im Klassenvergleich unterrepräsentiert. Die eigene Arbeitsbelastung und die schulische Situation werden kaum thematisiert. Während dieser Narrationsstil sich am seltensten auf die Schul- und Unterrichtsorganisation sowie auf das Lernverhalten und das eigene Erlebens von Schule bezieht, handelt es sich jedoch um Texte, die Corona am häufigsten global betrachten (75,6 %) sowie weitere soziale Probleme (40,4 %) und Folgen für weitere Teilsysteme der Gesellschaft (31,5 %) anführen. Die damit zum Ausdruck gebrachte Sensibilität gegenüber globalen Problemlagen geht mit der Hervorhebung von Solidarität als angemessener Form von Krisenbewältigung (17,8 %) einher. Lebenserfahrungen in und mit Krisendiskursen machen die besondere Sensibilität gegenüber sozialen Problemlagen verständlich. Die für diesen Narrationsstil charakteristische fehlende Artikulation von Zukunftsentwürfen lässt auf den starken Gegenwartseindruck schließen, den Krisen hinterlassen zu haben scheinen. Frühe Krisenerfahrungen und die ihnen zugrundeliegenden Diskurse könnten dies erklären lassen. Die Klasse 4 besteht zu 48,9 % aus Texten, die von Kindern verfasst wurden, die die Schule in Griechenland besuchen.

Alles fing so an: In China hatte ein Tier Corona-Virus und hat einem Menschen seine Krankheit übertragen. […] Ich habe an die Kinder die auf der Welt vor Hunger sterben gedacht. Die Luft hatte sich bereits wesentlich verbessert und mehrere Tiere trauten sich, [sich] sichtbar zu machen (UP 1026, MP 99,9 %, W).

## Zusammenfassung und Diskussion der Ergebnisse

Auf einem Kontinuum gesehen sind hier von der Einschätzung, es handle sich bei dieser Pandemie um einen zwar bedauerlichen, aber vorübergehenden ‚Zwischenfall‘ (Latenter Narrationsstil 3) und der Vorstellung, dieser durchaus akut bedrohliche Zustand werde ohne weitere Folgen in absehbarer Zeit ein Ende nehmen (Latenter Narrationsstil 2), über die Vorstellung von unabsehbaren (Spät‑)Folgen der Corona-Krise (Latenter Narrationsstil 1) bis hin zur Einstellung, der aktuelle Zustand sei – womöglich als Zeichen der sichtbar gewordenen Krisenhaftigkeit von Leben und Gesellschaft – womöglich ein Dauerzustand (Latenter Narrationsstil 4), alle graduellen Variationen vertreten.

Die identifizierten Narrationensstile können als Ausdruck für die unterschiedliche Verarbeitung des COVID-19 bedingten Zustandes verstanden werden: Sie verdeutlichen die unmittelbare Wahrnehmung der Schüler/innen sowie ihre subjektive Erfahrung von Umwälzungen in der eigenen Lebens- und Schulwelt, repräsentieren jedoch auch eine Lebenspraxis im Umgang mit Gegenwartsproblemen und ihrer Lösung in einer hypothetischen Welt.

Die Thematisierung von langfristigen Folgen der Corona-Pandemie im latenten Narrationsstil 1 geht mit der Imagination einer hypothetischen Zukunft einher. Die Fiktionalisierung ist Ausdruck einer Annäherung an das Unbekannte. Damit enthält die Klasse Elemente einer „routinenhaften Narration“ (vgl. auch zum Nachfolgenden Oevermann 2008) durch Konstruktion von verschiedenen Varianten einer möglichen Zukunft. Die Fähigkeit, aus der „traumatischen Krise“ neue Szenarios zu imaginieren ermöglicht es, die Diskontinuitäten in der Lebens- und Schulwelt als Anlässe für eine kreative Ausgestaltung der Zukunft zu sehen. Auffallend ist ein Verständnis lebensweltüberschreitender globaler Perspektiven sowie die Äußerung des Wunsches nach einer Krisenlösung durch Solidarität zwischen den Generationen. Der latente Narrationsstil 2 dagegen zeigt, dass die starke Wahrnehmung von Diskontinuitäten in der eigenen gegenwärtigen Lebenswelt diese lebensweltüberschreitenden Perspektiven verhindert, auch durch Stagnation auf der Fokussierung eigener Schwierigkeiten. Die Narrationen lesen sich als ‚Ventil‘ für die unmittelbare Konfrontation mit einem Zustand, dem die Schüler/innen aufgrund von fehlenden Gegenstrategien ausgeliefert scheinen. Dieser Narrationsstil verbleibt in der krisenhaften Narration des aktuellen Zustandes. Der latente Narrationsstil 3 unterscheidet sich dagegen durch eine distanziert erscheinende Wahrnehmung von Umwälzungen. Die Orientierung an die alte ‚Normalität‘ und die Konstruktion eines Ausgangs aus dem Krisenzustand und somit ihr Entwurf als ‚Zwischenfall‘ ist hier der Ausdruck der krisenhaften Narration. Im latenten Narrationsstil 4 werden kaum Diskontinuitäten zwischen der Lebens- und Schulwelt thematisiert. Vielmehr zeichnet sich diese Klasse durch eine andere Auffassung der „traumatischen Krise“ aus: Die Corona-Krise wird einer Reihe anderer globaler Probleme, die als bedeutendere Krisen betrachtet werden, nachgereiht. Die Nationalität (griechisch) erwies sich als signifikante Variable für die Klassenbildung. Eine mögliche Erklärung für die hohe Sensibilität gegenüber sozialen Problemen in diesem Narrationsstil könnte in der Kontextualisierung von Krisendiskursen und von damit einhergehenden Erfahrungen der Prekarisierung von individuellen Lebensverläufen während der sog. Wirtschaftskrise in der südeuropäischen Peripherie gesucht werden.

Schule als Institution wird in den einzelnen Klassen unterschiedlich akzentuiert dargestellt. Überwiegt in Klasse 1 eine Auffassung von Schule als zeitgebundene und veränderliche Einrichtung, scheint sich Schule in Klasse 3 als höchster Stabilitätsfaktor zu manifestieren, und trotz aller Krise(n) als Gewährsfigur für eine Rückkehr zur „Normalität“. In Klasse 2 dagegen wird Schule als adaptierungsfähige Organisation dargestellt und in Zusammenhang mit neuen – zeitlich limitierten – Regeln gebracht, die ihre Anpassungsnotwendigkeiten und -möglichkeiten demonstrieren.

Die verwendete Methodik in unserer Studie – so kann einschränkend hinzugefügt werden – zielte darauf, Narrationsstile zu identifizieren, wobei bewusst auf hypothesenprüfende Verfahren verzichtet wurde. Weiteres Potenzial zur Erweiterung dieser Untersuchung besteht in der Auswertung des gesamten Datenmaterials und Hinzuziehung weiterer Texte aus der zweiten Erhebungsphase (im Spätherbst/Winter 2020/21). Darüber hinaus wäre es interessant, Formen des je individuellen Umgangs mit den aus dem Spannungsverhältnis zwischen den analogen und digitalen Komponenten individueller Lern- und Lebenswelten resultierenden Krisenerfahrungen zu untersuchen. Dabei wäre zu prüfen, inwieweit Gegenwartsüberschreitung durch Antizipation von Entscheidungskrisen die Los-Lösung von der gegenwärtigen bedrohlichen COVID-Situation fördert und Formen der Krisenlösung begünstigt, die die Bewältigung von tiefgreifenden Umwälzungen erleichtern.

## Footnotes

1 Wenn wir in diesem Beitrag über die Corona-Zeit sprechen, beziehen wir uns auf die Phase der Schulschließungen sowie der ersten Wiedereröffnungen in der ersten Hälfte des Jahres 2020.

2 In Anlehnung an U. Oevermann (2008) verstehen wir den Begriff Krise im Verhältnis zur Routine. Krise bezeichnet keine Eigenschaft von Gegenständen, sondern ist immer in Relation zu einem prädikationsfähigen Subjekt zu verstehen. Krise charakterisiert eine Eigenschaft zwischen einem Gegenstand und einem Erfahrungssubjekt.

3 Gemeint ist hier vor allem die EU-Finanzkrise, die seit 2010 zu einer Prekarisierung der Lebens- und Arbeitsverhältnisse mit massiven Eingriffen in die sozialen und demokratischen Rechte für die griechische Bevölkerung geführt hat (vgl. dazu auch Fischer-Lescano 2013, S. 64).

4 Der mit der Software Recal3 (vgl. Freelon 2010) berechnete zufallskorrigierte Koeffizient Krippendorffs α bewegte sich variablenübergreifend zwischen 0,60 (Variable: *Erzähldynamik dynamisch*) und 0,93 (Variable: *Ende der Krise*); es lässt sich insgesamt von einer akzeptablen bzw. sehr guten Intercoder-Reliabilität sprechen.

5 Die Berechnung der LCA erfolgte mit der Software WINMIRA 2001. Die Häufigkeits- und Kontingenzberechnungen wurden mit dem Programm DYNAMIC (2.5) durchgeführt.

6 UP = Untersuchungsperson; MP = Membership Probability; W = weiblich, M = männlich.
